# Deposition of boron doped DLC films on TiNb and characterization of their mechanical properties and blood compatibility

**DOI:** 10.1080/14686996.2016.1262196

**Published:** 2017-01-16

**Authors:** Shahira Liza, Junko Hieda, Hiroki Akasaka, Naoto Ohtake, Yusuke Tsutsumi, Akiko Nagai, Takao Hanawa

**Affiliations:** ^a^ Department of Mechanical Sciences and Engineering, Tokyo Institute of Technology, Tokyo, Japan; ^b^ Institute of Biomaterials and Bioengineering, Tokyo Medical and Dental University, Tokyo, Japan; ^c^ Department of Mechanical Engineering, University of Malaya, Kuala Lumpur, Malaysia

**Keywords:** Boron doped DLC film, thin film, tribology, wettability, surface energy, platelet adhesion and activation, 10 Engineering and structural materials, 104 Carbon and related materials, 306 Thin film / Coatings

## Abstract

Diamond-like carbon (DLC) material is used in blood contacting devices as the surface coating material because of the antithrombogenicity behavior which helps to inhibit platelet adhesion and activation. In this study, DLC films were doped with boron during pulsed plasma chemical vapor deposition (CVD) to improve the blood compatibility. The ratio of boron to carbon (B/C) was varied from 0 to 0.4 in the film by adjusting the flow rate of trimethylboron and acetylene. Tribological tests indicated that boron doping with a low B/C ratio of 0.03 is beneficial for reducing friction (μ = 0.1), lowering hardness and slightly increasing wear rate compared to undoped DLC films. The B/C ratio in the film of 0.03 and 0.4 exhibited highly hydrophilic surface owing to their high wettability and high surface energy. An *in vitro* platelet adhesion experiment was conducted to compare the blood compatibility of TiNb substrates before and after coating with undoped and boron doped DLC. Films with highly hydrophilic surface enhanced the blood compatibility of TiNb, and the best results were obtained for DLC with the B/C ratio of 0.03. Boron doped DLC films are promising surface coatings for blood contacting devices.

## Graphical abstract

**Figure:**
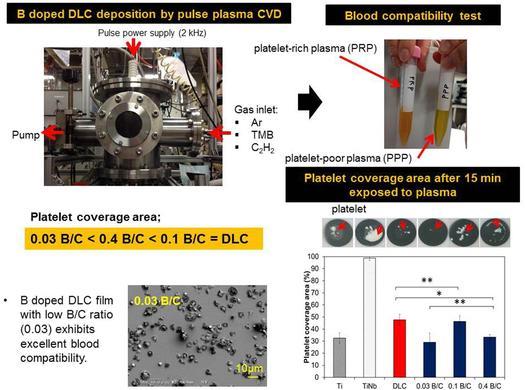

## 1. Introduction

Diamond-like carbon (DLC) films are a promising surface coating material for blood-contacting biomedical devices such as artificial hearts, artificial valves and stents, owing to their chemical inertness, excellent mechanical and tribological properties, and biocompatibility.[1–3] Biocompatibility can be considered in terms of blood compatibility and is often referred to as hemocompatibility. The interaction between circulating blood and the surface of biomaterials is related to blood compatibility. The blood compatibility of DLC films has been intensively investigated. The results showed that DLC films possess excellent compatibility with blood; they inhibit platelet adhesion and activation, thus reducing the risk of thromboembolism.[4, 5] However, one of the major limitations of DLC films is their poor adhesion on metal substrates due to their high internal stress.[6] The adhesive strength of the DLC films can be improved significantly by fabricating a thin interfacial layer of silicon between the DLC and the metal substrate,[7] by annealing,[8] or by element doping.[9]

Several works on boron incorporation into carbon films have indicated that boron can reduce the internal stress. [10–12] He et al. [12] reported that boron doped DLC with approximately 10 at.% of B (boron) exhibits 65% less stress than undoped DLC film. Previously, boron doped DLC films with 25.8 at.% of B was prepared by pulsed plasma chemical vapor deposition (CVD) on silicon substrates and their tribological performance was investigated.[13] The results exhibited tremendous boundary oil lubricated behavior for boron doped DLC film which has lower friction coefficient and wear rate than undoped DLC film. Low modulus of elasticity and pore growth under boundary lubrication conditions of film material properties are responsible for better performance. However, the use of a BC film as a blood-compatible coating on implants has not been explored yet. A study by Ahmad and Alsaad [14] only focused on enhancing the DLC-substrate adhesion strength through the use of boron as an additive to form a boron doped DLC films for bone fixation. Boron might have excellent biocompatibility properties, because of the wide range of uses of this element in the human body.[15–17]

Most blood contacting medical devices (such as a stent) are made from NiTi alloys.[18] However, several issues have limited the use of NiTi alloys, e.g. toxicity and corrosion. TiNb alloys have been found to be capable substitutions of NiTi alloys for biomedical blood contacting devices due to their lower cytotoxicity and higher corrosion resistance properties.[19, 20] McMahon et al. suggested that TiNb alloys can be used for stents because they exhibit the highest total recoverable strain among the Ti-based shape material alloys including NiTi alloys.[20]

Research on the corrosion behavior and biocompatibility of TiNb alloys are still in the progressive stage; however, it found very rear to study on the blood compatibility of TiNb alloys. Hence, it is essential to evaluate the blood compatibility of coated and uncoated TiNb alloy material for stents. The blood compatibility of uncoated and coated TiNb alloy was investigated by an *in vitro* platelet adhesion experiment where Ti alloy was used as the control material.

## 2. Experimental details

This study was divided into two parts to evaluate the potential of boron doped DLC films as a coating for blood-contacting equipment such as stents. The first part of the study was focused on the characterization of deposited film on silicon substrate. This part has been performed to understand the mechanical and tribological behavior of boron doped DLC before the stent implantation. Their surface properties such as surface roughness and wettability were also studied. These characterizations are considered important in determining coating properties which are critical to their development. In the second part, the blood compatibility of boron doped DLC films deposited on TiNb alloy was evaluated.

### 2.1. Film deposition

Conventional DLC and boron doped DLC films were prepared on (100) silicon and TiNb substrates using pulsed plasma CVD. CVD technique was used because the deposition process involves depositing a boron doped DLC from a gaseous phase. The surfaces of biomedical TiNb (23 at.% Nb) and Ti grade 2 (JIS H 4600) substrates with a diameter of 10 mm and thickness of 1.5 mm were ground by abrasive paper (100 to 2000 grit) to remove surface defects and contaminants. The TiNb and Ti substrates were subsequently polished by water milling to obtain a mirror surface. Finally, all substrates were cleaned through the ultrasonic process with the help of distilled water, methanol, and acetone for 40, 20 and 20 min respectively. The polished and cleaned substrates were loaded into a deposition chamber by positioning on a sample holder. A turbomolecular pump was used to evacuate the chamber and maintained the background pressure below 4.0 × 10^−7^ Pa. To generate plasma, a 14 kHz monopolar pulsed power was applied and a 2.1 W input pulsed power was used for 44.2 μs for film deposition. Before deposition, the substrate surfaces were sputter-cleaned by Ar (Argon) plasma for 2 h when the gas flow rate was maintained at 30 cm^3^ min^–1^ and voltage of –3 k. C_2_H_2_ was used for DLC films deposition and a mixture of C_2_H_2_ and B(CH_3_)_3_ (trimethylboron, TMB) were used for boron doped DLC film deposition. TMB was selected as a boron source because of its lower toxicity than other boron sources.[21] Boron doped DLC films with different boron contents were deposited by changing the flow rate ratio of C_2_H_2_ to TMB. The boron doped DLC film with the highest boron content was prepared using TMB only. In this study, a DLC interlayer was employed between the boron doped DLC film with the highest boron content and the silicon substrate used in the tribological test to prevent oxidation-induced delamination. In our previous study, we reported that this film consists of pores and that the diffusion of moisture through the pores can cause delamination.[13] To improve the film adhesion, an interlayer was deposited between the DLC and boron doped DLC and the TiNb substrate using tetramethylsilane (TMS). The deposition conditions are shown in Table 1.

**Table 1.** Pulsed plasma CVD conditions.

Deposition parameter	DLC	B-DLC1	B-DLC2	B-DLC3	DLC interlayer	TMS interlayer
C_2_H_2_ gas flow rate (cm^3^ min^–1^)	20	20	20	–	20	–
TMB gas flow rate (cm^3^ min^–1^)	–	3	10	15	–	–
TMS gas flow rate (cm^3^ min^–1^)					–	10
Pressure (Pa)	3	3	3	5	3	3
Bias voltage (kV)	−3	−3	−3	−3	−3	−2.5
Deposition time (h)	3	3	3	3	2	0.2

### 2.2. Film composition

XPS analysis of sample surfaces were carried out with ESCA-1700R (Physical Electronics Co., Inc, MN, USA) equipment for observing the chemical composition and bonding states where Al Kα is used as an X-ray source. The spectral regions measured were C1s and B1s. A combination of Gaussian and Lorentzian curve fitting techniques was employed to carry out the best fit. The chemical bonding of the films was characterized by Fourier transform infrared (FTIR) spectroscopy with the help of FT/IR-4200 (JASCO International Co., Ltd, Tokyo, Japan).

### 2.3. Evaluation of mechanical and tribological properties

A maximum indentation depth approach (less than 10% of coating thickness) was employed to measure the film hardness and Young’s modulus with the help of a PICODENTOR HM-500 equipment from Fischer Instruments K.K. (Saitama, Japan). The measurement was accomplished at 100 dissimilar location and the indentation load was used 0.8 mN for the 1–1.8 μm-thick deposited film. The DLC films are frequently utilized as coating purpose and forms a tribo-system with carbon steel material in biomedical devices. Thus the wear and friction characteristics of direct contact between steel and DLC was investigated at dry condition with the help of a ball-on-disk tribometer (S-DLC1).[22] In the ball-on-disk sliding tests, a steel ball was utilized and the modulus of elasticity and Vickers hardness were found 210 GPa and 800 HV, respectively for stainless steel ball of 6 mm diameter. A normal load of 1 N was applied to the ball while passing against the film. This experiment was conducted at ambient conditions (i.e. 32–56% relative humidity and 25°C) and the sliding speed was maintained at 0.209 m s^−1^. The time taken for each run experiment was about 250 min for 100,000 cycles. A VK-9700 (KEYENCE, NJ, USA) 3D laser scanning microscope was employed to observe the cross sectional area of the worn track and the wear volume loss was calculated. Later, the wear rate was determined as the ratio of wear volume and the sliding distance.

### 2.4. Surface roughness, contact angle, and surface energy

SPA300 (SII Seiko Instruments Co., Ltd, Chiba, Japan) atomic force microscopy (AFM) instrument was used to analyze the surface roughness (*R*_*a*_) of the sample. On the surface, 10 different locations in a 2 μm × 2 μm area was chosen for measuring the *R*_*a*_ values. The corresponding standard deviation were derived from the 10 replicates and the average value was presented as the result.

The assessment of static contact angles of droplets of distilled water, hexadecane and diiodomethane (1 μl each) on the sample surfaces refers to the wettability of each film. Image of the droplets were captured by a digital camera. The contact angles were measured using ImageJ software.[23] Four different locations on the surface were considered for measuring the angle and the average was presented as a result. An unpaired *t-*test was performed to find the significant level where *p* < 0.05 was reflected statistically significant. In accordance with Kitazaki and Hata,[24, 25] the extended Fowkes theory has been used to calculate the surface energy of the samples from the measured contact angles. The surface energy of the liquids used in the calculation are shown in Table 2.

**Table 2.** Surface energy of the liquids used in calculation [26, 27].

Liquid	Surface energy (mN m^–1^)			
	ϒ^d^	ϒ^p^	ϒ^h^	ϒ^T^
Distilled water	29.1	1.3	42.4	72.8
Hexadecane	27.9	0	0	27.9
Diiodomethane	46.8	4.0	0	50.8

ϒ^d^: dispersion force component, ϒ^p^: polar force component, ϒ^h^: hydrogen-bonding force component, ϒ^T^: total surface energy.

### 2.5. *In vitro* platelet adhesion experiments

This research work has been approved by the Research Ethics Committee of the Institute of Biomaterials and Bioengineering, Tokyo Medical and Dental University (no. 7, 26 March 2013). The subjects of this research have signed an informed consent form. The *in vitro* short-term platelet adhesion experiment protocol was in accordance with the procedure reported by Tanaka et al. [28] Fresh blood plasma (20 ml) was collected from a healthy adult. Anticoagulant blood was prepared by mixing fresh human blood with sodium citrate. The sodium citrate concentration was 3.8 wt.%, and the volume ratio of the sodium citrate and the fresh blood was 1:9. Centrifugation was performed at 900 rpm (140 g) for 15 min using a centrifuge (2410, Kubota Corp., Osaka, Japan) to obtain a platelet-rich plasma (PRP). Subsequently, a part of the PRP was centrifuged for 15 min at 2900 rpm (1370 g) to obtain platelet-poor plasma (PPP). The density of the platelets was adjusted in the PRP to 1 × 10^5^ μl^–1^ by dilution with the PPP. 0.25 mol l^–1^ of CaCl_2_ solution was added to the PRP for platelet activation. Before the experiment the samples were ultrasonically cleaned in ethanol for 10 min and incubated for 30 min at 37°C. On the top of the samples, 2 μl of the PRP was seeded and incubation was carried out at 37°C for 5, 10, and 15 min. During incubation, some of the platelets interacted with and adhered to the surface. After that, the samples were washed with phosphate-buffered saline (PBS; pH 7.4) until all unconglutinated platelets were removed. The adherent platelets were then fixed for 2 h at room temperature in 2 wt.% glutaraldehyde solution. After fixation, the samples were washed and dehydrated three times in a graded ethanol series (30, 50, 70, 90, 99.5%) for 15 min each then in 99.5% ethanol dehydrated by sodium sulfate for 8 min. Then, the specimens were dried by supercritical drying using CO_2_. Finally, the specimens were examined by optical microscopy (OM) and scanning electron microscopy (SEM). For the SEM observation, dry samples were coated with gold. The number and the morphology of adherent platelets were measured to determine the blood compatibility of the surfaces. The platelet coverage per unit area (850 μm × 550 μm) was investigated via binary imaging using computer-aided ImageJ analysis software. Measurement was performed at 10 randomly selected areas on each surface. The number of adherent platelets is expressed as the percentage platelet coverage area and the standard error. Values were compared statistically by an unpaired *t*-test. Results with *p* < 0.05 were considered to be statistically significant. Several researchers used the same approach in order to study the blood compatibility of deposited film.[29–31]

## 3. Results and discussion

### 3.1. Chemical compositions and bonding

Table 3 lists the atomic content, the B/C ratio, and the binding energy of the C1s and B1s peaks of the deposited films obtained from XPS analysis. The boron content in the boron doped DLC films increased to 2.6 and 9.2 at.% when the TMB flow rate was increased to 3 and 10 cm^3^ min^–1^, respectively, with a constant gas flow rate of C_2_H_2_. The chemical composition of amorphous boron carbide film deposited using only TMB was 25.8 at.% boron, 60.2 at.% carbon and 14 at.% oxygen.[13] The atomic concentration of boron for this film is consistent with the chemical formula of TMB, that is, B(CH_3_)_3_. However, this film had a high oxygen concentration due to the oxidation process, which generally occurs during the deposition and post-deposition. The B/C ratio was calculated from the atomic composition percentages of B and C.

**Table 3.** Chemical composition and binding energy of deposited films obtained from XPS analysis.

Sample	Gas flow rate ratio (C_2_H_2_:TMB)	Binding energy /eV				B/C ratio
		C (%)	Peak position	B (%)	Peak position	
DLC	20:0	100	284.1	–	–	0
B-DLC1	20:3	97.4	284.0	2.6	188.9	0.03
B-DLC2	20:10	90.8	283.8	9.2	189.3	0.1
B-DLC3^[^^13^^]^	0:15	60.2	284.7	25.8	191.2	0.4

In accordance with the calculation, boron doped DLC films deposited in this study are hereafter categorized as having B/C ratio of 0.03, 0.1 and 0.4. The binding energy information of the C1s and B1s peaks obtained by XPS analysis is shown in Table 3. It was observed that the binding energy of the main C1s peak of the deposited films slightly decreased from 284.1 to 283.8 eV with increasing boron content, while the binding energy of the main B1s peak increased from 188.9 to 189.3 eV. The shift of the C1s peak to a lower binding energy with increasing boron content can be attributed to the occurrence of C-B bonding and an increase in *sp* ^2^ bonding.[32, 33] This result indicates that the boron incorporated in DLC in this study changed the structure of the DLC film. For the boron doped DLC film with a B/C ratio of 0.4, the main C1s and B1s peaks are at 284.7 and 191.2 eV, respectively. This finding indicates that the structure of this film is different from that of the boron doped DLC films with B/C ratios of 0.03 and 0.1 owing to the deposition of TMB only.

**Figure 1.**
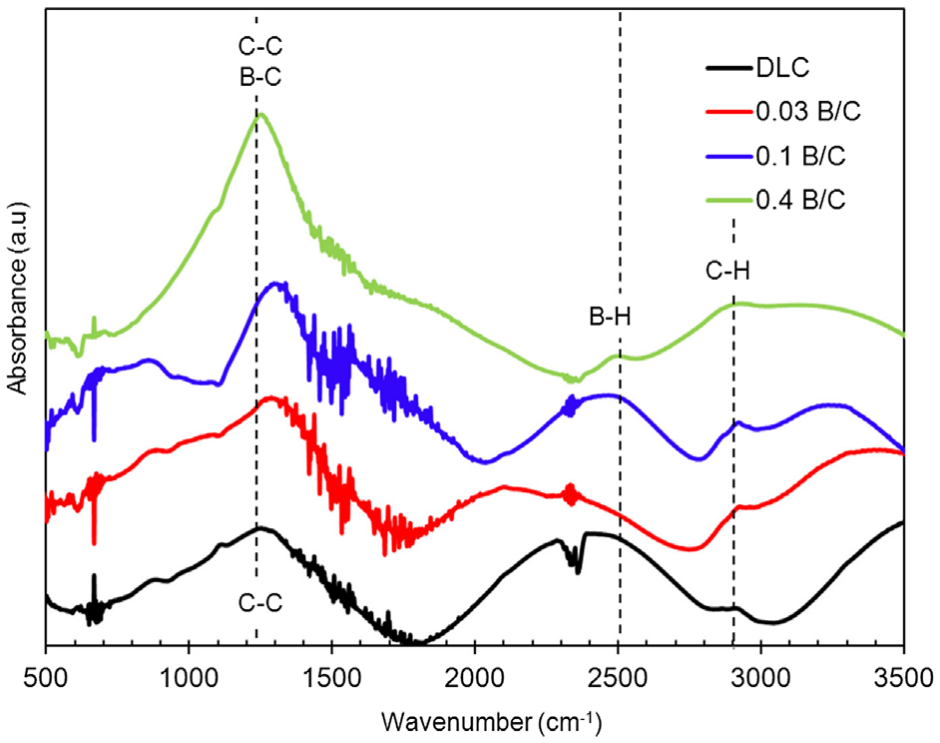
IR spectra of DLC film and boron doped DLC films with B/C = 0.03, 0.1 and 0.4.

Further analysis was carried out by FTIR spectroscopy to determine the bonding structure of carbon, hydrogen, and boron atoms in the boron doped DLC films. The spectra of the deposited samples obtained from FTIR are shown in Figure 1. There the fingerprint region (1350 to 1700 cm^−1^) contains overlapped peaks that were assigned as follows: 800–880 cm^−1^ to the C–H out-of-plane bending, 1100–1400 cm^−1^ to the C–H_x_ bending mode, 1400–1600 cm^−1^ to the aromatic *sp* ^2^ C stretching modes and 1500–1700 cm^−1^ to the olefinic *sp* ^2^ C stretching modes.[35] Another strong absorption band at 1250–1280 cm^−1^ is attributed to the B-C stretching modes.[36]

In this study, C–C *sp* ^3^ bonds could not be detected by IR absorption because they have a very small dipole moment, and were likely swamped by multiple overlapping peaks in the fingerprint region. Meanwhile, the absorption band around 2900 cm^−1^ was attributed to CH stretching modes.[36] The weak absorption band of the B–H bond appears at a wavenumber of approximately 2500 cm^−1^.[34]

### 3.2. Surface roughness, contact angle, and surface energy

**Figure 2.**
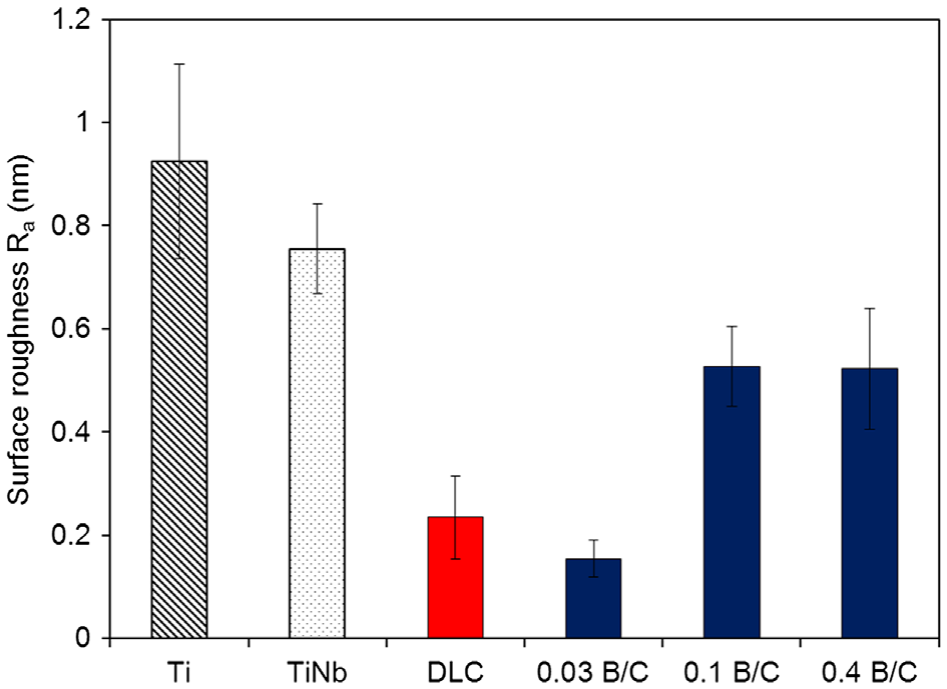
Surface roughness (*R*_*a*_) of Ti, TiNb and DLC films and boron doped DLC films with B/C = 0.03, 0.1, and 0.4.

**Figure 3.**
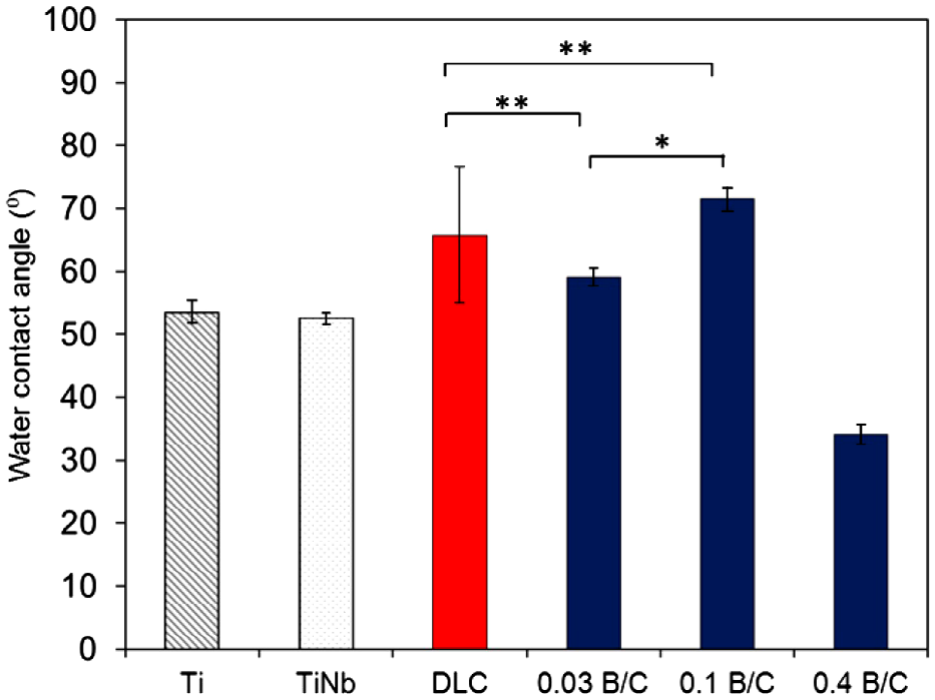
Water contact angle of Ti, TiNb, and DLC films and boron doped DLC films with B/C = 0.03, 0.1, and 0.4. * denotes a significant difference between B/C = 0.03 and B/C = 0.1 (*p < 0.05). ** denotes no significant difference between DLC, B/C = 0.03 and B/C = 0.1 (**p > 0.05).

Figure 2 displays the surface roughness (*R*_*a*_) of the Ti, TiNb, and DLC films and the boron doped DLC films obtained from AFM measurement. The results show that the boron doped DLC film with B/C = 0.03 had the smoothest surface. The water contact angle was measured and the surface energy was calculated to determine the hydrophilic properties of the films. Figure 3 shows the obtained contact angles with distilled water for six different samples including uncoated Ti and TiNb substrates. The results indicate that all the samples in this study had hydrophilic surfaces. It was also observed that the DLC film and the boron doped DLC films with B/C = 0.03 and 0.1 had higher contact angles than the Ti and TiNb substrates. The boron doped DLC film with B/C = 0.4 exhibited the lowest contact angle, indicating its highest wettability among the samples. The surface energy of the deposited films was calculated to investigate the factors that affect the wettability of the deposited films.

**Figure 4.**
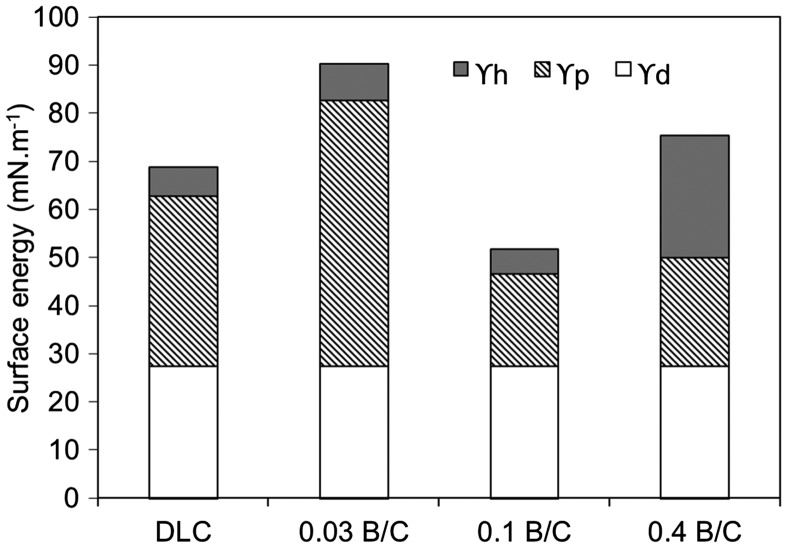
Surface energy of DLC film and boron doped DLC films with B/C = 0.03, 0.1, and 0.4.

The total surface energy of the deposited films can be expressed as the sum of the dispersion component (ϒ^d^), the polar component (ϒ^p^), and the hydrogen bonding component (ϒ^h^). Figure 4 shows the total surface energy of the DLC and boron doped DLC films coated on a TiNb substrate. The total surface energy of the boron doped DLC film with B/C = 0.03 was highest among the deposited films. The large polar contribution was found to be the dominant factor contributing to the high surface energy for this film. Different forces and interactions between atoms and molecules at the interface such as hydrogen bonds, covalent bonds and dipole–dipole interactions have determined the polar component.[31, 37] Agathopoulos and Nikolopoulos [38] suggested that the larger the contribution of the polar component to the surface energy, the greater the tendency of the surface to attract polar liquids and the higher the hydrophilicity. According to the IR spectra, this film consisted of B–C bonds which are known to be polar bonds with a hydrophilic characteristic. The hydrogen bonding component was also found to play a minor role in the higher surface energy of the boron doped DLC film with B/C = 0.03 than those of the DLC film and the boron doped DLC film with B/C = 0.1. The latter two films had a low hydrogen bonding component owing to the presence of C–H bonds in both films and B–H bonds in the film with B/C = 0.1 according to the IR spectra. These are known to be nonpolar bonds as they cannot form hydrogen bonds. This finding indicates that the boron doped DLC film with B/C = 0.1 had a hydrophobic characteristic that resulted in lower wettability (*p* < 0.05) and a lower surface energy than the film with B/C = 0.03. In addition, the DLC film was also hydrophobic characteristic owing to its low surface energy, although its wettability was similar (*p* > 0.05) to that of the film with B/C = 0.03. For the film with B/C = 0.4, the hydrogen bonding component made a major contribution to the surface energy. The larger hydrogen bonding component is due to the presence of B–O bonds on the surface, as confirmed from the XPS analysis, which easily form hydrogen bonds with polar water. Overall, the results indicate that the wettability of the deposited films was determined by the chemical structure at the surface.

### 3.3. Mechanical and tribological properties

**Figure 5.**
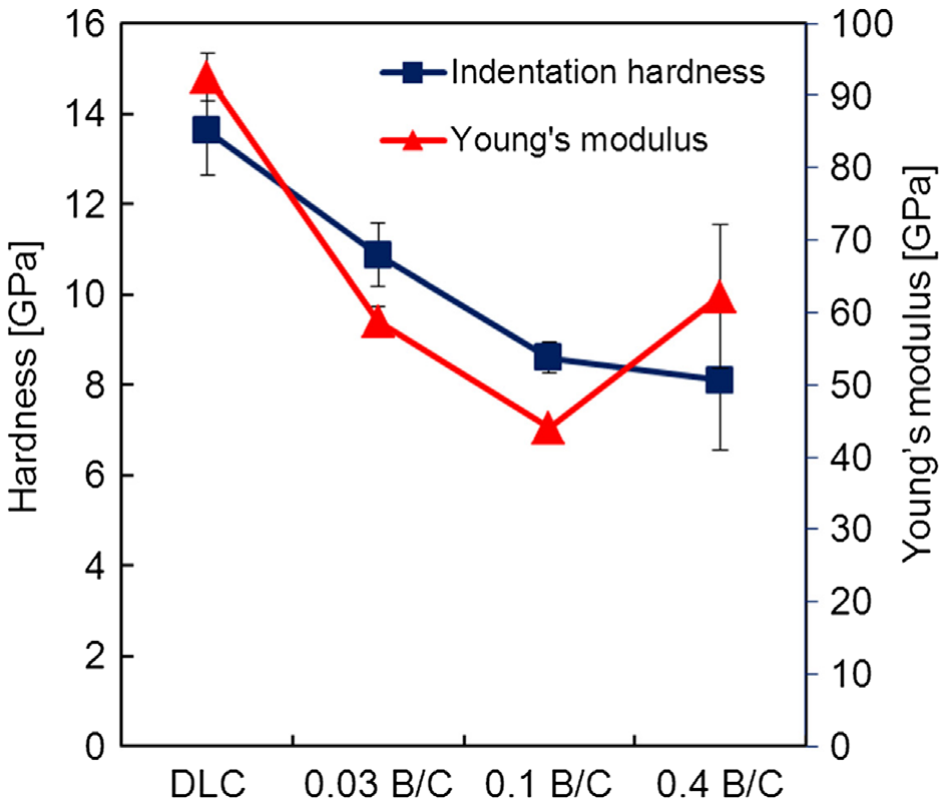
Indentation hardness and Young’s modulus for DLC film and boron doped DLC films with B/C = 0.03, 0.1, and 0.4.

Figure 5 shows the hardness and Young’s modulus of the DLC film and the boron doped DLC films as a function of their B/C ratio. The DLC film without B exhibited the highest hardness of about 13.6 GPa and a Young’s modulus of about 92.5 GPa. As the B/C ratio increased to 0.03, the hardness and Young’s modulus decreased to 10.9 and 58.8 GPa, respectively. For the highest B/C ratio of 0.4, the hardness was about 8.1 GPa. The Young’s modulus decreased to 44 GPa for the film with B/C = 0.1 and increased to of 62.2 GPa at B/C = 0.4. This may have been due to the different chemical bonding configuration of the boron doped DLC film with B/C = 0.4 resulting from the deposition of TMB only. Decreases in the hardness and Young’s modulus with increasing boron content are usually associated with the reduced effect of boron on the coordination in the carbon network.[11]

**Figure 6.**
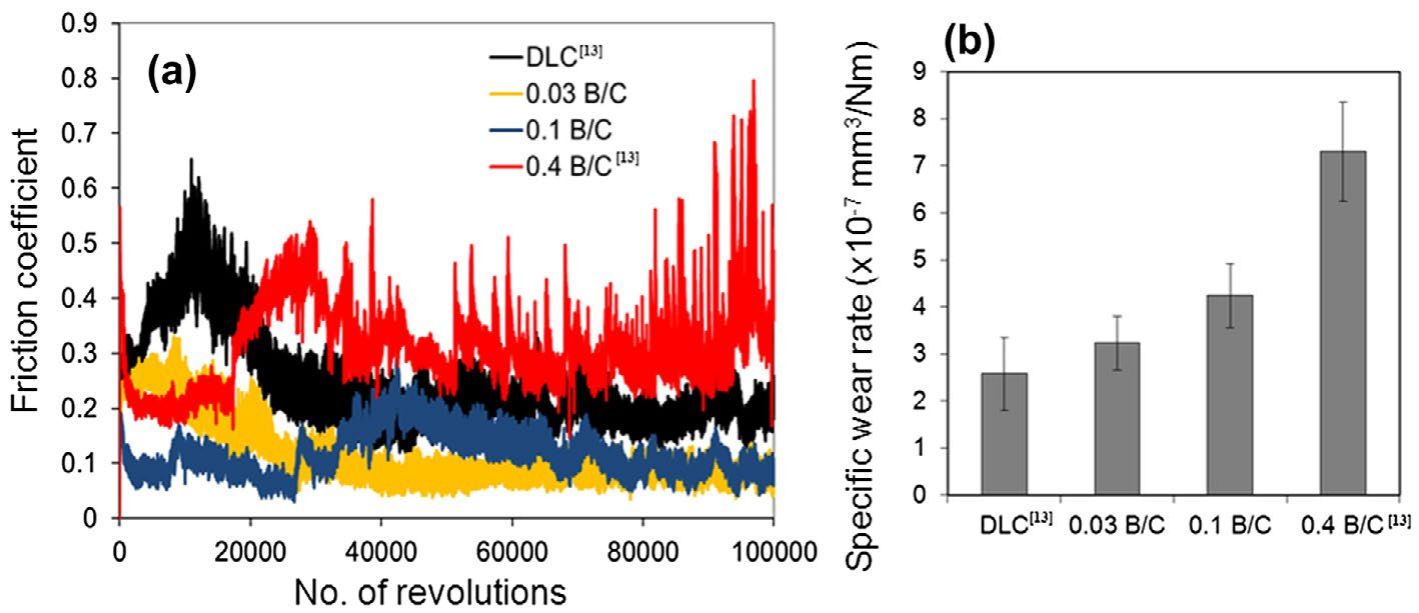
(a) Friction coefficient and (b) wear rate of DLC and boron doped DLC films after sliding against steel ball under dry condition.

Figure 6 shows the friction coefficients and wear rates of the DLC and boron doped DLC films obtained from the ball-on-disk tests. Figure 6(a) shows the friction coefficients of the DLC film and the boron doped DLC films with different B/C ratios under a dry condition. All the boron doped DLC films exhibited a lower friction coefficient than the DLC film, including the multilayer film with B/C=0.4, at which our previous study showed the boron doped DLC layer was completely removed after 20,000 sliding cycles.[13] The film with the low B/C ratio of 0.03 exhibits the lowest friction coefficient. The friction coefficient was 0.25 after a running-in period of approximately 10,000 sliding cycles, then decreased to a steady-state value of 0.1. In our previous study, we claimed that boron doped DLC films have a lower friction coefficient than DLC under a dry condition owing to the lower pressure in the contact area due to the low modulus of elasticity and the effect of the transfer layer attached to the steel counter face, which can change the tribological properties significantly.[13] The results for the friction coefficient in this study indicate that the film with B/C = 0.03 is beneficial for reducing friction despite its slightly higher wear rate than the DLC film. The wear rate of the films increased with the boron content as shown in Figure 6(b). This expected behavior was due to the lower indentation hardness, as described above, which gives rise to greater material loss related to the ductile failure induced by the Hertzian nature of the contact.[39]

In this study, it was difficult to relate the friction and wear with wettability or surface energy of the deposited samples because the tribological tests have been conducted in a dry condition. However, this relationship can be easily explained by referring to our previous study where the tribological tests were conducted under oil boundary lubricated conditions.[13] The results of wettability measurements indicate that all deposited films had hydrophilic surfaces, including the DLC films in this study. Dresselhuis et al..[40] mentioned that oil wets hydrophobic surfaces much better than hydrophilic surfaces. Therefore, oil should be a better lubricant for hydrophobic than for hydrophilic surfaces. During the tribology test, when the flat film surface is hydrophilic, the layer of oil which is initially on the film has been immediately taken away. So in this case the oil does not make a layer which is capable to reduce the friction. However, in this study boron doped DLC with B/C = 0.4 exhibited pores on the film surface. Thus, pores can act as oil reservoirs in the tribological system, which prevent fluid film breakage and result in a low friction coefficient.

### 3.4. Platelet adhesion

Platelet adhesion and activation on foreign material surfaces play a central role in the thrombosis of blood-contacting medical devices. Therefore, platelet adhesion and activation must be assessed to determine the blood compatibility of a new material. In this study, the quantity and morphology of platelets on the surfaces of the deposited films were examined as to determine the blood compatibility of the deposited films.

**Figure 7.**
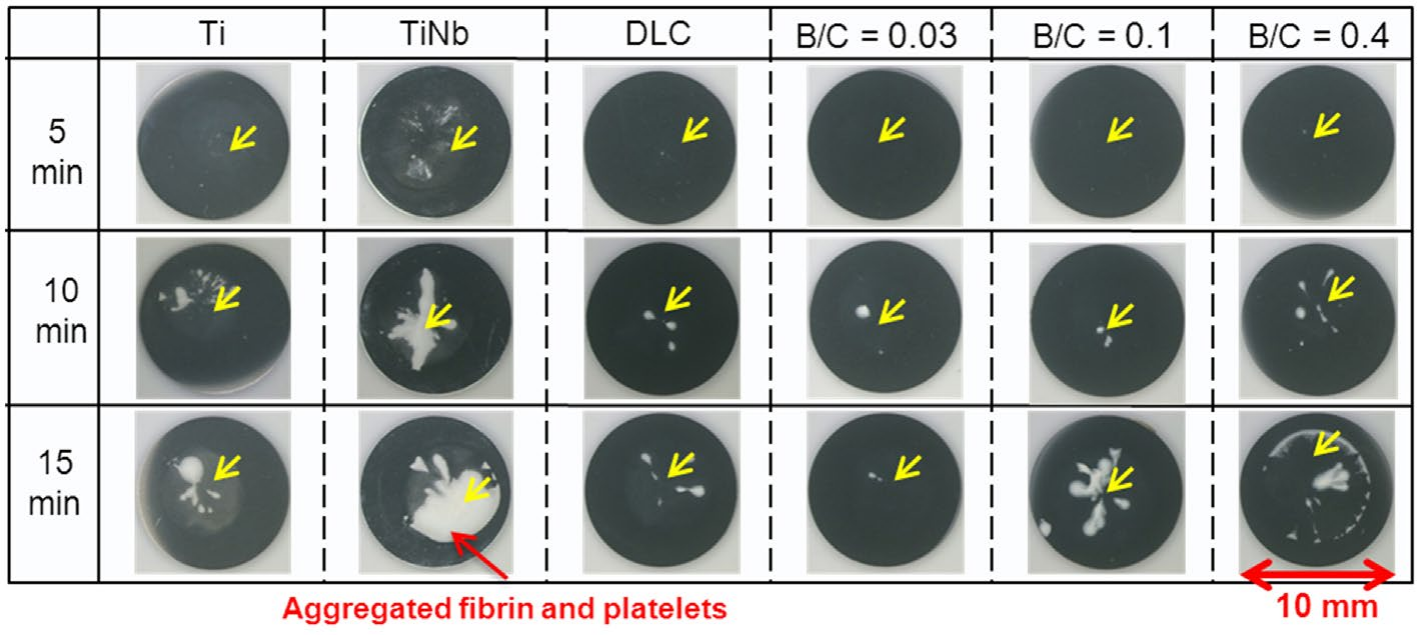
Photographs of platelets on surfaces of uncoated Ti and TiNb substrates and DLC and boron doped DLC films coated on TiNb substrates.

Figure 7 shows photographs of platelets on the surfaces of uncoated Ti and TiNb substrates, and DLC and boron doped DLC films coated on TiNb substrates after incubation times of 5, 10, and 15 min. The aggregated fibrin and platelets on the surface of uncoated TiNb alloy are predominant compared with on the other samples after a 15 min incubation time. The DLC and boron doped DLC films coated on TiNb substrates improved the blood compatibility of the TiNb substrates by suppressing the aggregation of fibrin and platelets on the film surfaces. It is clearly shown that the boron doped DLC film with B/C = 0.03 coated on a TiNb substrate exhibited the least amount of aggregated fibrin and platelets.

**Figure 8.**
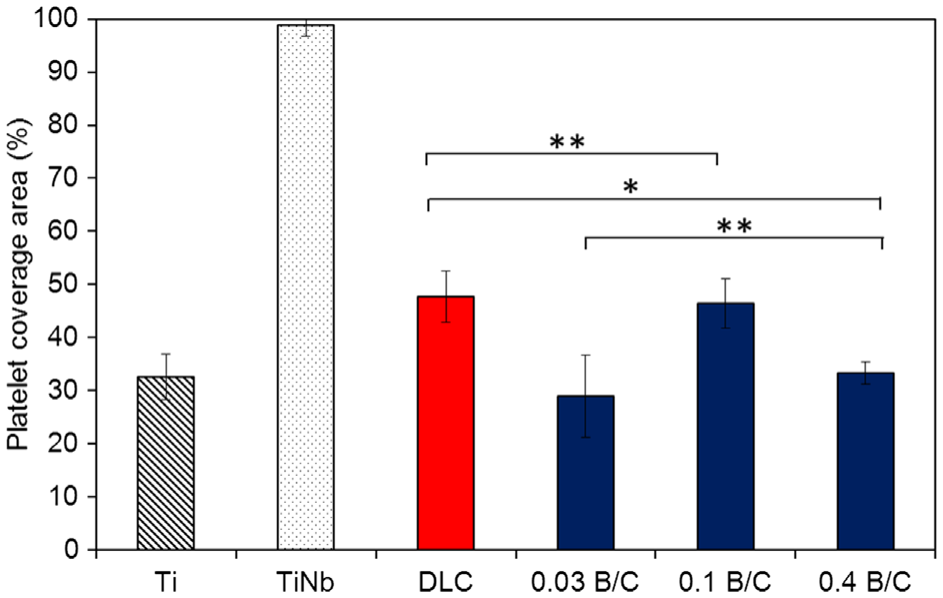
Platelet coverage area measured on the surfaces of uncoated Ti and TiNb substrates, and DLC and boron doped DLC films coated on TiNb substrates after incubation for 15 min. * denotes a significant difference between DLC and B/C = 0.4 (*p < 0.05). ** denotes no significant difference between DLC and B/C = 0.1, and B/C = 0.03 and 0.4 (**p > 0.05).

**Figure 9.**
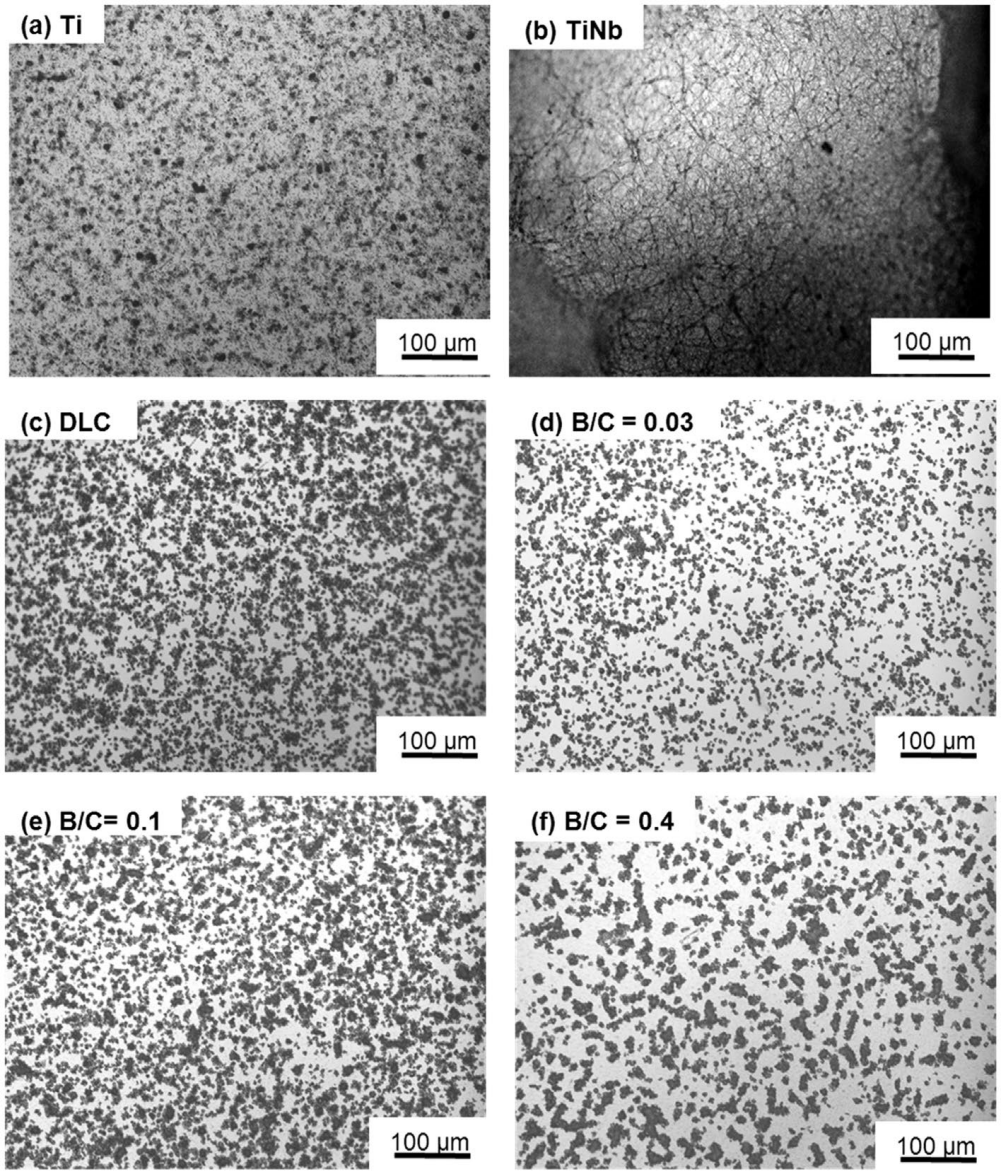
Platelet coverage on the surfaces of uncoated Ti and TiNb substrates and DLC and boron doped DLC films coated on TiNb substrates after incubation for 15 min.

Figure 8 displays the coverage area of aggregated fibrin and platelets and adherent and activated platelets on the sample surfaces observed by optical microscopy (binary image) over an area 850 μm × 550 μm (Figure 9) after 15 min incubation. The platelet coverage areas were manually calculated using ImageJ analysis software in the common area for adherent platelets on the surface of the deposited films, as indicated by yellow arrows in Figure 8.[23] In Figure 9, the black area is characterized as adherent platelets for all samples except for TiNb, for which aggregated fibrin and platelets covered almost the entire surface. The values in Figure 8 represent averages of 10 measurements. This result clearly demonstrated that the coverage areas of aggregated fibrin and platelets for the deposited films on TiNb substrates were significantly smaller than those for the uncoated TiNb substrates. It was observed that the boron doped DLC films with B/C = 0.03 and 0.4 exhibited similar coverage areas of adherent and activated platelets (*p* > 0.05). The DLC film and the boron doped DLC film with B/C = 0.1 also had similar percentages of adherent platelets (*p* > 0.05). However, the coverage areas of adherent platelets for the boron doped DLC films with B/C = 0.03 and 0.4 were lower than those of the DLC film and the film with B/C = 0.1 (*p* < 0.05). The coverage areas of adherent platelets for the deposited films can be summarized as B/C of 0.03 = B/C of 0.4 < DLC = B/C of 0.1.

**Figure 10.**
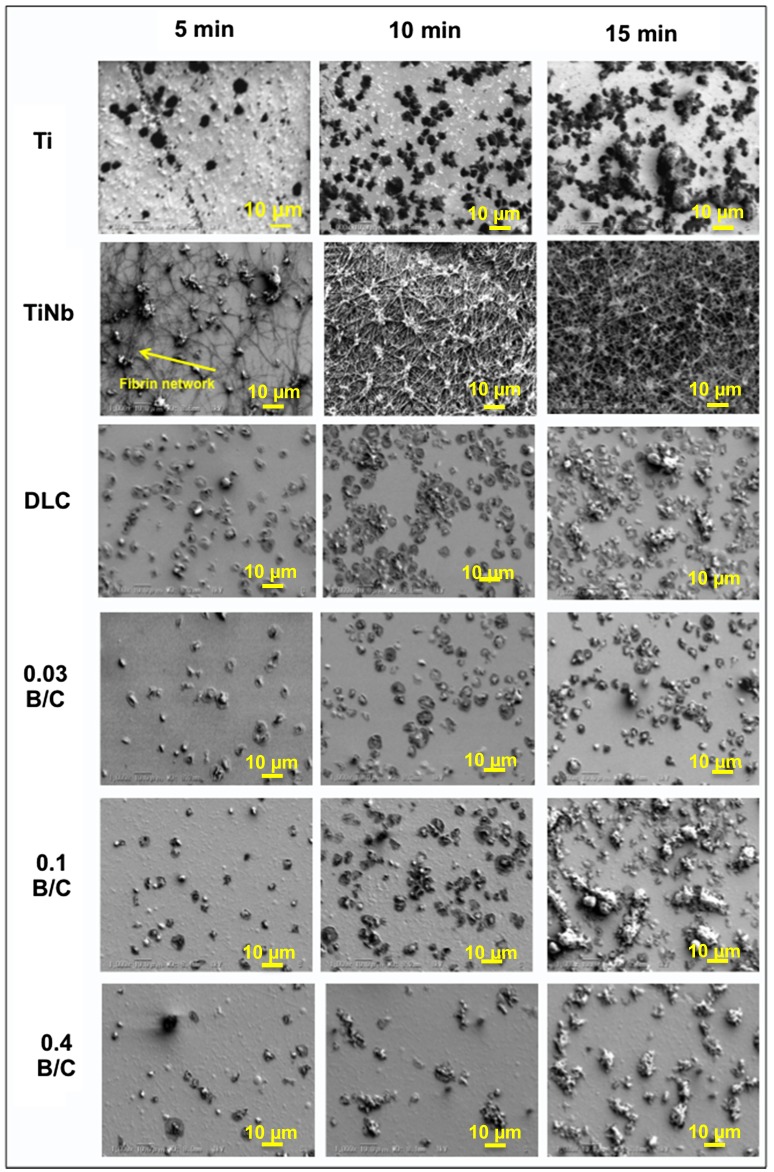
SEM images of platelets adhered on surface of uncoated Ti and TiNb substrates and DLC and boron doped DLC films coated on TiNb substrates.

Figure 10 shows SEM images of the platelets adhered to the uncoated Ti and TiNb substrates and the DLC and boron doped DLC films coated on TiNb surfaces after incubation times of 5, 10, and 15 min. The SEM images were captured on surfaces with common patterns of adherent platelets as indicated by the yellow arrows in Figure 7. We observed that the white substance present in Figure 7 was the platelets and fibrinogen. From the SEM observation, the activation of platelets can be judged from the changes in their morphology. A structural change is believed to lead to activation promoting the thrombus cascade process. During activation, the platelets attach to the sample surface and lose their round shape, form pseudopodia, and spread over the surface.[41, 42] This then leads to fibrin network formation and the subsequent aggregation of fibrin and platelets to form a thrombus.

In the present study, a large number of activated platelets with a certain degree of spreading were observed on all the sample surfaces. For the TiNb surfaces, a large fibrin network was found after 5 min incubation. After 10 min incubation, the aggregation of fibrin and platelets was clearly observed with all the platelets linked with each other. After 15 min, the aggregated fibrin and platelets appeared to be thicker than after 10 min incubation. For the DLC and boron doped DLC films, the spreading and circularity of the activated platelets in the form of pseudopodia were observed after incubation for 15 min. However, the adherent platelets on the surface of the film with B/C = 0.03 were nearly round and had fewer pseudopodium states. In comparison, the platelets that spread over the surfaces of the other deposited films had pseudopodium states. Note that, among the deposited films, the film with B/C = 0.03 provided the least platelet activation, indicating the lowest risk of thrombus formation. From the SEM observation, the platelet adhesion and activation levels can be summarized as B/C of 0.03 < B/C of 0.4 < DLC = B/C of 0.1.

From the coverage ratio of the aggregated fibrin and the morphology of the adherent platelets, the boron doped DLC films with B/C = 0.03 and 0.4 exhibited better blood compatibility than the DLC film and the film with B/C = 0.1. However, the platelet activation morphology indicated that the activated platelets on the film with B/C = 0.03 were nearly round with less pronounced pseudopodium states, while the film with B/C = 0.4 exhibited activated platelets that spread and pseudopodium states. From this observation, it is suggested that the film with B/C = 0.03 had better blood compatibility than the film with B/C = 0.4.

Many studies have demonstrated the factors that affect the blood compatibility of biomaterials, which include surface roughness, wettability and surface charge density [43–46] Our approach in this study involves the characterization of surface properties such as surface roughness, wettability, and surface energy that influence the blood compatibility of the deposited films. However, the surface roughness of the deposited films is not considered to be an important factor determining blood compatibility because it is at the nanoscale (below 100 nm).[46]

In the previous discussion, the films with B/C = 0.03 and 0.4 were characterized as having the highest hydrophilicity among the deposited films. This finding indicates better blood compatibility can be attributed to high hydrophilicity. The correlation between hydrophilicity and the reduction of platelet adhesion has been confirmed in several studies [43–46]

Plasma protein adsorption is the first event that occurs when a foreign material comes into contact with blood, which affects the subsequent blood-material interactions.[47] The adhesion of fibrinogen from blood plasma plays an important role in the coagulation cascade.[48, 49] Fibrinogen has been reported to be the main plasma protein mediating the adhesion of platelets.[50] The affinity of fibrinogen was indeed reported to be higher and more rapid for CH_3_ groups (hydrophobic) than for OH groups (hydrophilic).[51] Thus, the amount of adherent fibrinogen is higher on hydrophobic surfaces than on hydrophilic surfaces.[51, 52]

Charge–transfer interactions are also important in protein adsorption and can determine the blood compatibility of biomaterials. Fibrinogen has the same electronic structure as a semiconductor, and electron transfer from its occupied valence band into the free states of a material surface may cause thrombus formation.[53] On the other hand, a hydrophilic surface consists of hydroxyl groups (OH), which are electrically negative terminal groups. Such a hydrophilic surface has been shown to prevent platelet adhesion by providing an oxide layer that acts as a semiconductor that insulates and prevents electron conduction at a material surface.[54]

Note the special blood compatibility properties for the boron doped DLC film with B/C = 0.4, which had a different chemical structure from the other tested boron doped DLC films because it was deposited using TMB gas only. In comparison the film with B/C = 0.1, although the film with B/C = 0.4 had a higher boron content, it had fewer adherent platelets. According to the previous discussion, this film exhibited higher hydrophilicity than the film with B/C = 0.1 owing to it consisting of oxide constituents as proven by XPS analysis which led to a low number of adherent platelets. In our previous study, we reported that a boron doped DLC film with B/C = 0.4 had a porous surface.[13] Koh et al. [55] described how geometrical features in addition to the specific surface chemistry have a significant effect on reducing platelet adherence. Another study by Sutherland et al. [56] suggested that in the early phase of platelet exposure (approximately 10 min), the platelet binding rate is controlled by the nanoscale surface topography (40 nm diameter and 10 nm depth) rather than the surface chemistry, which increases platelet binding. However, according to the results of our SEM observation in Figure 8, fewer platelets were observed after 5 and 10 min incubation on the surface of the film B/C = 0.4 than on the other films, in contrast to the study by Sutherland et al. [56] Hence, we believe that owing the effect of the special features on the surface of the film with B/C = 0.4 on blood compatibility can be neglected owing to its nanoscale pore size. Further study is required to confirm the negligible effect of pores on the blood compatibility of the film with B/C = 0.4.

The synthesized boron doped DLC film appears to be a potential new surface modification for blood-contacting devices such as stents. As mentioned in the Introduction, TiNb alloys are reported to be an alternative to NiTi alloys as material for stents due to their lower cytotoxicity and high corrosion resistance.[19, 20] However, this study has proved that the uncoated TiNb substrates exhibit aggregated of fibrin and platelet after incubation for 15 min. On the other hand, the DLC and boron doped DLC films coated on TiNb substrates exhibit least platelet activation compare with uncoated TiNb substrates, indicating low risk of thrombus formation. Note that, the addition of boron in carbon film (2.6 at.% B) improves the blood compatibility due to low platelet adherent and activation compared to DLC. These observations support the present suggestion that boron doped DLC with B/C ratio of 0.03 (2.6 at.% B) may be the optimum composition for an antithrombogenic surface on a blood-contacting device.

We believe that boron doping of DLC films is appropriate and safe for their use in biomedical applications. The biocompatibility of boron-containing materials has been addressed by several researchers. Bahl et al. [57] studied Ti-6Al-4 V alloy doped with 0.1 wt.% boron. Cells were well spread on the doped alloy indicating a good biocompatibility. Weber et al. [58] implanted boron into the surface of Ni-Ti shape memory alloys used in stents. The acceptable boron concentration in stents was 0.005–0.5 wt.%. Based on these references, we believe that boron doped DLC films can be safely used in biomedical applications, especially at low boron concentrations such as 0.03 B/C (2.6 at.% B).

## 4. Conclusions

In this study, we prepared boron doped DLC films with different B/C ratios (0 to 0.4) by controlling the flow rate of TMB in the reaction gas mixture with C_2_H_2_. The nanoindentation hardness was found to decrease from 13.6 to 8.1 GPa with increasing B/C ratio. The Young’s modulus decreased as the B/C ratio increased except for the film with B/C = 0.4. From the mechanical and tribological evaluation, the results indicated that DLC has a better performance than boron doped DLC film except for the friction coefficient (μ = 0.1 for the film with B/C = 0.03 and μ = 0.2 for DLC film). These results may suggest that DLC film can increase the useful life of the stents. Boron doped films with B/C ratios of 0.03 exhibited a better blood compatibility than undoped DLC films and uncoated Ti and TiNb substrates. Such films can suppress the thrombus generation, which is a major problem after stent placement and can trigger life-threatening device failures. In conclusion, B/C = 0.03 is the optimum B/C ratio for boron doped DLC coatings in blood-contacting devices.

## Disclosure statement

No potential conflict of interest was reported by the authors.

## References

[CIT0001] Hang R, Qi Y (2010). A study of biotribological behavior of DLC coatings and its influence to human serum albumin. Diamond Relat Mater.

[CIT0002] Sui JH, Gao ZY, Cai W (2007). DLC films fabricated by plasma immersion ion implantation and deposition on the NiTi alloys for improving their corrosion resistance and biocompatibility. Mater Sci Eng A.

[CIT0003] Gutensohn K, Beythien C, Bau J (2000). In Vitro analyses of diamond-like carbon coated stents: reduction of metal ion release, platelet activation, and thrombogenicity. Thromb Res.

[CIT0004] Jones MI, McColl IR, Grant DM (2000). Protein adsorption and platelet attachment and activation, on TiN, TiC, and DLC coatings on titanium for cardiovascular applications. J Biomed Mater Res.

[CIT0005] Sui JH, Cai W (2006). Effect of diamond-like carbon (DLC) on the properties of the NiTi alloys. Diamond Relat Mater.

[CIT0006] Takeno T, Shiota H, Sugawara T (2009). Highly adherent tungsten-containing diamond-like carbon (W-DLC) coating on a NiTi shape memory alloy under 10% tensile strain. Diamond Relat Mater.

[CIT0007] Bonetti LF, Capote G, Santos LV (2006). Adhesion studies of diamond-like carbon films deposited on Ti6Al4 V substrate with a silicon interlayer. Thin Solid Films.

[CIT0008] Ferrari AC, Kleinsorge B, Morrison NA (1999). Stress reduction and bond stability during thermal annealing of tetrahedral amorphous carbon. J Appl Phys.

[CIT0009] Tang XS, Wang HJ, Feng L (2014). Mo-doped DLC nanocomposite coatings with improved mechanical and blood compatibility properties. Appl Surf Sci.

[CIT0010] Chhowalla M, Yin Y, Amaratunga GAJ (1996). Highly tetrahedral amorphous carbon films with low stress. Appl Phys Lett.

[CIT0011] Tan M, Zhu J, Han J (2007). Stress evolution of tetrahedral amorphous carbon upon boron incorporation. Scr Mater.

[CIT0012] He XM, Walter KC, Nastasi M (2000). Plasma-immersion ion-processed boron-doped diamond-like carbon films. J Phys: Condens Matter.

[CIT0013] Liza S, Ohtake N, Akasaka H (2015). Tribological and thermal stability study of nanoporous amorphous boron carbide films prepared by pulsed plasma chemical vapor deposition. Sci Technol Adv Mater.

[CIT0014] Ahmad AA, Alsaad AM (2007). Adhesive B-doped DLC films on biomedical alloys used for bone fixation. Bull Mater Sci.

[CIT0015] Koga K, Kaji A, Hirosaki K (2006). Cytotoxic evaluation of cubic boron nitride in human origin cultured cells. Toxicol in Vitro.

[CIT0016] Klepper CC, Williams JM, Truhan JJ (2008). Tribo-mechanical properties of thin boron coatings deposited on polished cobalt alloy surfaces for orthopedic applications. Thin Solid Films.

[CIT0017] Lousinian S, Kalfagiannis N, Logothetidis S (2009). Optical and surface characterization of amorphous boron nitride thin films for use as blood compatible coatings. Solid State Sci.

[CIT0018] Rebelo N, Fu R, Lawrenchuk M (2009). Study of a nitinol stent deployed into anatomically accurate artery geometry and subjected to realistic service loading. J Mater Eng Perform.

[CIT0019] Wang YB, Zheng YF (2009). Corrosion behaviour and biocompatibility evaluation of low modulus Ti-16Nb shape memory alloy as potential biomaterial. Mater Lett.

[CIT0020] McMahon RE, Ma J, Verkhoturov SV (2012). A comparative study of the cytotoxicity and corrosion resistance of nickel-titanium and titanium-niobium shape memory alloys. Acta Biomater.

[CIT0021] Cifre J, Puigdollers J, Polo MC (1994). Trimethylboron doping of CVD diamond thin films. Diamond Relat Mater.

[CIT0022] Tiainen V-M (2001). Amorphous carbon as a bio-mechanical coating-mechanical properties and biological applications. Diamond Relat Mater.

[CIT0023] Ferreira T, Rasband W (2012). ImageJ user guide.

[CIT0024] Kitazaki Y, Hata T (1972). Surface-chemical criteria for optimum adhesion. J Adhes.

[CIT0025] Hata T, Kitazaki Y, Saito T (1987). Estimation of the surface energy of polymer solids. J Adhes.

[CIT0026] Černe L, Simončič B, Željko M (2008). The influence of repellent coatings on surface free energy of glass plate and cotton fabric. Appl Surf Sci.

[CIT0027] Jongwannasiri C, Moolsradoo N, Khantachawana A (2012). The comparison of biocompatibility properties between ti alloys and fluorinated diamond-like carbon films. Adv Mater Sci Eng.

[CIT0028] Tanaka Y, Kurashima K, Saito H (2009). In vitro short term platelet adhesion on various metals. *J Artif Organs*.

[CIT0029] Hasebe T, Nagashima S, Kamijo A (2013). Hydrophobicity and non-thrombogenicity of nanoscale dual rough surface coated with fluorine-incorporated diamond-like carbon films: Biomimetic surface for blood-contacting medical devices. Diamond Relat Mater.

[CIT0030] Saito T, Hasebe T, Yohena S (2005). Antithrombogenicity of fluorinated diamond-like carbon films. Diamond Relat Mater.

[CIT0031] Roy RK, Choi HW, Yi JW (2009). Hemocompatibility of surface-modified, silicon-incorporated, diamond-like carbon films. Acta Biomater.

[CIT0032] Rusop M, Tian XM, Kinugawa T (2004). The effects of boron content in the target of pulsed laser deposition on the properties of boron doped amorphous carbon thin films. Mod Phys Lett B.

[CIT0033] Pu J-C, Wang S-F, Lin C-L (2010). Characterization of boron-doped diamond-like carbon prepared by radio frequency sputtering. Thin Solid Films.

[CIT0034] Onate JI, Garcia A, Bellido V (1991). Deposition of hydrogenated B-C thin films and their mechanical and chemical characterization. Surf Coat Technol.

[CIT0035] Yu J, Wang EG, Ahn J (2000). Turbostratic boron carbonitride films produced by bias-assisted hot filament chemical vapor deposition. J Appl Phys.

[CIT0036] Wada Y, Yap YK, Yoshimura M (2010). The control of B-N and B-C bonds in BCN ﬁlms synthesized using pulsed laser. Diamond Relat Mater.

[CIT0037] Bendavid A, Martin PJ, Randeniya L (2009). The properties of fluorine containing diamond-like carbon films prepared by plasma-enhanced chemical vapor deposition. Diamond Relat Mater.

[CIT0038] Agathopoulos S, Nikolopoulos P (1995). Wettability and interfacial interactions in bioceramic-body-liquid systems. J Biomed Mater Res.

[CIT0039] Liu SB, Peyronnel A, Wang QJ (2005). An extension of the Hertz theory for three-dimensional coated bodies. Tribol Lett.

[CIT0040] Dresselhuis DM, Klok HJ, Stuart MAC (2007). Tribology of o/w emulsions under mouth-like conditions: determinants of friction. Food Biophys.

[CIT0041] Goodman SL, Grasel TG, Cooper SL (1989). Platelet shape change and cytoskeletal reorganization on polyurethaneureas. J Biomed Mater Res.

[CIT0042] Allen RD, Zacharski LR, Widirstky ST (1979). Transformation and motility of human platelets: details of the shape change and release reaction observed by optical and electron microscopy. J Cell Biol.

[CIT0043] Liu H, Zhang D, Shen F (2012). Hemocompatibility and anti-endothelialization of copper-titanium coating for vena cava filters. Surf Coat Technol.

[CIT0044] Hancock LF, Fagan SM, Ziolo MS (2000). Hydrophilic, semipermeable membranes fabricated with poly(ethylene oxide)-polysulfone block copolymer. Biomaterials.

[CIT0045] Wan GJ, Yang P, Shi XJ (2005). In vitro investigation of hemocompatibility of hydrophilic SiNx: H films fabricated by plasma-enhanced chemical vapor deposition. Surf Coat Technol.

[CIT0046] Hasebe T, Ishimaru T, Kamijo A (2007). Effects of surface roughness on anti-thrombogenicity of diamond-like carbon films. Diamond Relat Mater.

[CIT0047] Vroman L, Adams AL (1969). Effect of heparin on reactions at aminated polymer-blood interfaces. J Colloid Interface Sci.

[CIT0048] Brash JL, Davidson VJ (1976). Adsorption on glass and polyethylene from solutions of fibrinogen and albumin. Thromb Res.

[CIT0049] Massa TM, McClung WG, Yang ML (2007). Fibrinogen adsorption and platelet lysis characterization of fluorinated surface-modified polyetherurethanes. J Biomed Mater Res A.

[CIT0050] Tsai WB, Grunkemeier JM, McFarland CD (2002). Platelet adhesion to polystyrene-based surfaces preadsorbed with plasmas selectively depleted in fibrinogen, fibronectin, vitronectin, or von Willebrand’s factor. J Biomed Mater Res A.

[CIT0051] Roach P, Farrar D, Perryl CC (2005). Interpretation of protein adsorption: surface-induced conformational changes. J Am Chem Soc.

[CIT0052] Slack SM, Horbett TA (1992). Changes in fibrinogen adsorbed to segmented polyurethanes and hydroxyethylmethacrylate-ethylmethacrylate copolymers. J Biomed Mater Res.

[CIT0053] Hashmi MSJ (2014). Comprehensive Materials Processing.

[CIT0054] Huang N, Yang P, Leng YX (2003). Hemocompatibility of titanium oxide films. Biomaterials.

[CIT0055] Koh LB, Rodriguez I, Venkatraman SS (2010). The effect of topography of polymer surfaces on platelet adhesion. Biomaterials.

[CIT0056] Sutherland DS, Broberg M, Nygren H (2001). Influence of nano-scale surface topography and chemistry on the functional behavior of an adsorbed model macromolecule. Macromol Biosci.

[CIT0057] Bahl S, Raj S, Vanamali S, Suwas S, Chatterjee K (2014). Effect of boron addition and processing of Ti–6Al–4 V on corrosion behaviour and biocompatibility. Maters Techno Adv Biomaters.

[CIT0058] Weber J, O’brien B Boron-enhanced shape memory endoprostheses.

